# Intervertebral disc degeneration associated with vertebral marrow fat, assessed using quantitative magnetic resonance imaging

**DOI:** 10.1007/s00256-020-03419-7

**Published:** 2020-05-28

**Authors:** Yayun Ji, Weifeng Hong, Mouyuan Liu, Yuying Liang, YongYan Deng, Liheng Ma

**Affiliations:** Department of Medical Imaging, The First Affiliated Hospital of Guangdong Pharmaceutical University, Guangzhou City, 510080 Guangdong Province China

**Keywords:** Lumbar disc degeneration, Quantitative MRI, Bone marrow fat

## Abstract

**Objective:**

To investigate the potential clinical application of quantitative MRI in assessing the correlation between lumbar vertebrae bone marrow fat deposition and intervertebral disc degeneration.

**Materials and methods:**

A total of 104 chronic lower-back pain volunteers underwent 3.0-T MRI with T2-weighted imaging, T2 mapping, and iterative decomposition of water and fat with echo asymmetry and least squares estimation (IDEAL-IQ) between August 2018 and June 2019. Each disc was assessed with T2 value by T2 mapping, and the L1-S1 vertebral bone marrow fat fraction was assessed by IDEAL-IQ. The differences and relationship between T2 value and the adjacent vertebral bone marrow fat fraction values within the five Pfirrmann groups, five age groups, and five lumbar levels were statistically analyzed.

**Results:**

The vertebral bone marrow fat fraction had a significant negative correlation with T2 values of nucleus pulposus’ T2 values (*p* < 0.001). However, the significant negative correlation was only found between T2 values of nucleus pulposus and adjacent vertebral bone marrow fat in Pfirrmann II–III, L1/2-L5/S1 level, and 40–49 years’ age groups. Pfirrmann grades of the intervertebral disc were positively correlated with adjacent vertebrae bone marrow fat fraction (*p* < 0.05).

**Conclusion:**

Lumbar bone marrow fat deposition significantly increases during the early stages of intervertebral disc degeneration. Quantitative measurements of bone marrow fat deposition and water content of intervertebral discs have a predictive value and are an important supplement to the qualitative traditional classification strategies for the early stages of intervertebral disc degeneration.

## Introduction

Lumbar intervertebral disc degeneration (IVDD) is a common orthopedics’ disease that causes lower-back pain [1–3], with early biochemical changes which include proteoglycans and hydration loss, and late morphologic changes which include disc height loss, nucleus pulposus (NP) herniation, and annular tears [4]. The lumbar disc is the largest avascular tissue within the human body, nourished primarily by the diffusion of micro-vessels in the adjacent vertebral bodies through the cartilaginous endplate [5]. Interference with this pathway may be a risk factor for intervertebral disc (IVD) metabolism. The conversion of the hematopoietic bone marrow to bone marrow fat (BMF) accompanies the decrease of vertebral perfusion and nutrition supplies [6, 7]. However, whether the adjacent vertebra marrow fat can affect IVDD remains unknown.

Osteoporosis is closely associated with IVDD [8] while vertebral osteoporosis is associated with IVDD in postmenopausal women [9]. IVDD is often concomitant with osteoporosis [10, 11], suggesting that osteoporosis and IVDD development may be an accompanying process and may explain why postmenopausal women experience more back pain than men [12]. Studies have reported the possibility of delaying IVDD progression by improving bone metabolism and vertebral osteoporosis. For example, sodium alendronate delays the progression of lumbar IVDD in ovariectomized rats by improving bone quality [13].

Folman et al. showed that bone marrow density (BMD) positively correlated with disc degeneration [14]. One explanation is that most studies measure BMD using dual-energy X-ray absorptiometry (DXA). Since the DXA measurement of BMD often interferes with the results due to osteophyte formation, it may not accurately reflect the degree of bone changes in the vertebral body. Other non-invasive methods are required to accurately detect and explore vertebral BMD changes.

Vertebral BMF deposition is negatively associated with bone density and closely related to osteoporosis [15, 16]. During osteoporosis, the differentiation programs that yield osteoblasts and adipocytes from mesenchymal stem cells within the marrow favor adipogenesis to osteoblastogenesis leading to increased marrow fat with reduced bone formation [17–19]. Various advanced MRI techniques have been used to measure BMF content, and IDEAL-IQ was confirmed as one of the most convenient and accurate techniques to quantify BMF [20, 21]. T2 mapping, a novel quantitative technique, evaluates disc degeneration. Compared with traditional Pfirrmann classification, T2 mapping is less affected by subjective factors, and the measurement of disc degeneration is more accurate [22, 23]. This study investigated whether BMF parameters correlate with IVDD at different ages by quantitative MRI.

## Materials and methods

### Study population

This cross-sectional cohort study was approved by the Ethics Committee of our hospital. All subjects provided written informed consent before examination. A total of 104 male and female volunteers of different age groups (20–29 years, 30–39 years, 40–49 years, 50–59 years, 60–70 years) with chronic lower-back pain between August 2018 and June 2019 were recruited in this study. Among them, 47 were male (mean age 44.6 years) and 56 were female (mean age 44.3 years). The inclusion criteria included the following: (1) male or female volunteers with chronic lower-back pain (lasted for more than 3 months), which was based on Alleva et al.’s study [24]; (2) underwent routine MRI lumbar spine examination, IDEAL-IQ, and T2 mapping imaging with satisfactory image quality. Exclusion criteria included the following: (1) medical history of diseases, such as a tumor, spondylolisthesis, lumbar scoliosis, lumbar trauma or fracture, surgery, spinal infectious diseases, hemopathy, and metabolic bone disease; (2) incomplete imaging data or poor image quality.

### MRI imaging protocol

MRI was performed using a 3T system (GE Discovery MR750 3.0T, America) with a dedicated spine coil. The lumbar spine scanning sequence included routine T1WI and T2WI sequence, IDEAL-IQ, and T2 mapping sequence. The following imaging parameters were used: (1) Sagittal T1WI (TR/TE = 809/11 ms), T2WI (TR/TE = 3093/120 ms), transversal T2WI (TR/TE = 3500/120 ms), slice thickness 3 mm, intersection gap 0.4, FOV 320 × 320 mm, matrix 132 × 132, the number of excitations (NEX) 2; (2) Sagittal IDEAL-IQ:TR 10.5 ms, minimum TE 1.2 ms, maximum TE 6.5 ms, FOV 300.0 mm × 300.0 mm, matrix 132 × 132, layer thickness 5.0 mm, acquisition time 168 s; (3) transversal T2 mapping TR 718 ms, TE1-TE8 time 9.7 ms, 19.4 ms, 29.1 ms, 38.7 ms, 48.4 ms, 58.1 ms, 67.8 ms, and 77.5 ms, layer thickness 3 mm, layer spacing 1 mm, NEX 1.

### Quantitative MR image analysis

**Fig. 1:**
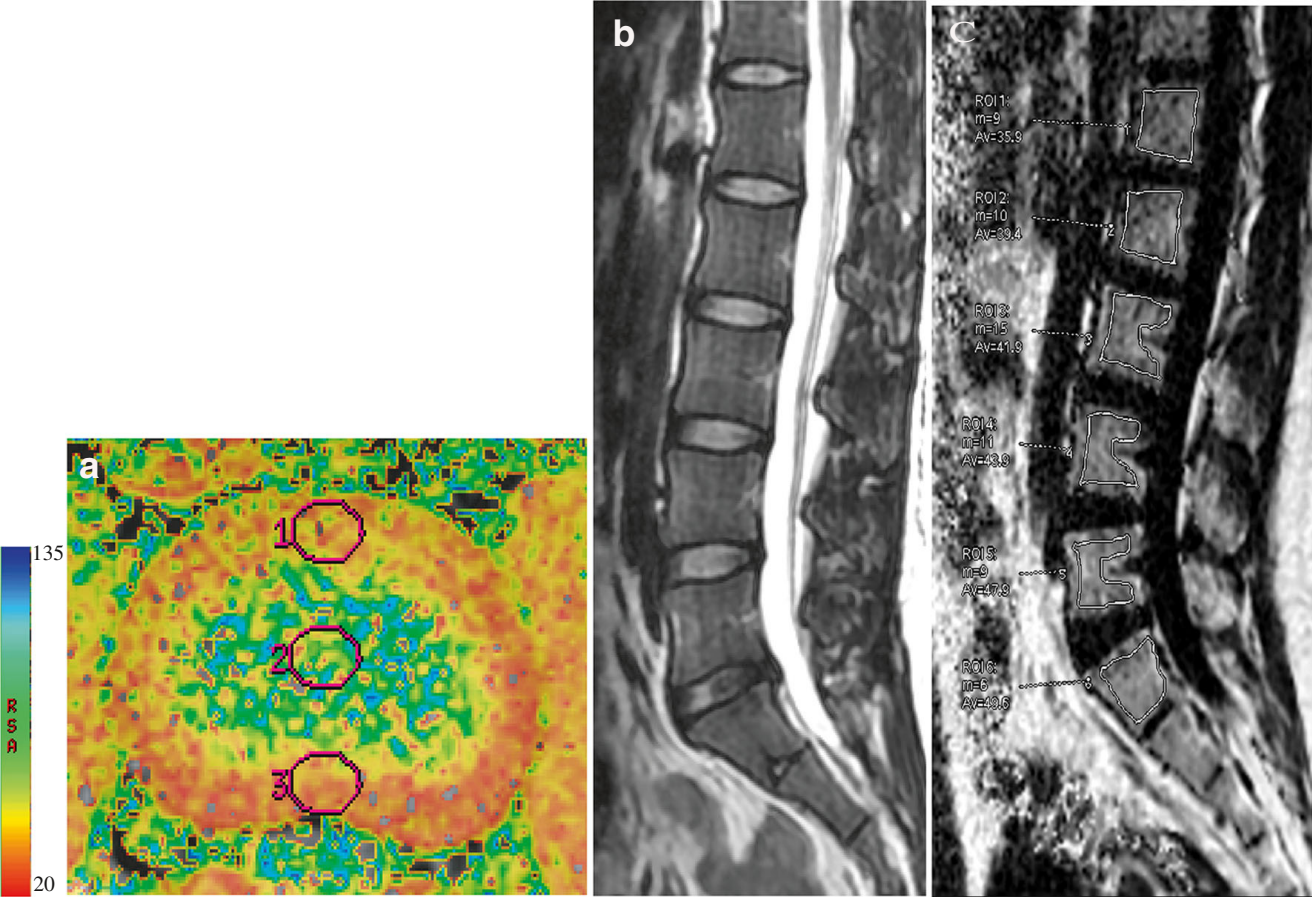
T2WI was used to assess the Pfirrmann classification. The ROIs were drawn in the axial T2 mapping images referred to the sagittal view to measure the T2 values of nucleus pulposus (NP), anterior annulus fibrosus (AAF), and posterior annulus fibrosus (PAF) separately (1 = AAF, 2 = NP, 3 = PAF). The vertebral bone marrow fat was measured with ROI in vertebrae, in which the endplate and basivertebral foramen were avoided

All routine MR images were used to identify pre-existing abnormalities of the lumbar vertebrae, and T2WI was used to assess the Pfirrmann classification [25]; the specific classification standards are listed in Table 1; we excluded 23 Pfirrmann V IVDs. All vertebral bone marrow fat fractions (FF) were measured along with the remaining 497 Pfirrmann I–IV IVDs’ T2 values on the AW4.6 workstation. All MRI images were analyzed by two experienced radiologists in clinical diagnostic and spine MRI research, MLH, and JYY. We loaded FatFrac images in the REFOMAT system and measured the FF value of the vertebral bone marrow (VFF). The L1-S1 vertebral body was mapped manually at the three central layers, to avoid the endplate and basivertebral foramen. To avoid bias, the average value of the three measurements was considered (Fig. 1). We defined the fat fraction of adjacent superior vertebral bodies as SFF and the fat fraction of adjacent inferior vertebral bodies as IFF. The Functool post-processing was also performed on the T2 mapping images. We indicated the round region of interesting (ROI) within the anterior annulus fibrosus (AAF) to measure the T2 value and copied the ROI onto the nucleus pulposus (NP) and posterior annulus fibrosus (PAF) to measure the T2 values, respectively (Fig. 1). The T2 values of the NP, AAF, and PAF of L1/2-L5/S1 discs were all measured. The final Pfirrmann results were obtained by consensus of the two experts, and the average value of the two expert measurements was taken for the quantitative result.

**Table 1:** Classification of disc degeneration [25]

Pfirrmann classification	Structure	Nucleus and anulus	Signal intensity	Height of intervertebral disc
I	Homogeneous, bright white	Clear	Hyperintense, isointense to cerebrospinal fluid	Normal
II	Inhomogeneous with or without horizontal bands	Clear	Hyperintense, isointense to cerebrospinal fluid	Normal
III	Inhomogeneous, gray	Unclear	Intermediate	Normal to slightly decreased
IV	Inhomogeneous, gray to black	Lost	Intermediate to hypointense	Normal to moderately decreased
V	Inhomogeneous, black	Lost	Hypointense	Collapsed disc space

### Statistical analysis

Agreement between the two experts’ Pfirrmann gradings was evaluated using Cohen’s kappa (*κ*) statistic. The strength classification based on kappa values was as follows: excellent (*κ* = 0.8–1.0); substantial (*κ* = 0.6–0.80); moderate (*κ* = 0.4–0.6); fair (*κ* = 0.2–0.4); and slight (*κ* = 0.0–0.2). A Bland–Altman plot was used to analyze the quantitative measurements between the two experts, by plotting the mean of the two measurements against their difference with a 95% limits of agreement (LOA) (= mean difference ± 1.96 × SD of the difference). Bivariate correlations of the T2 value, Pfirrmann I–V in IVD, and FF in the adjacent vertebra were measured using the Pearson product-moment two-tailed correlation coefficient analysis. The T2 values and the adjacent SFF/IFF value differences within the five Pfirrmann groups, five age groups, and five lumbar levels were examined using the Kruskal–Wallis test [26]. Analyses, where the overall test was significant, and pairwise comparisons were carried out using the Mann–Whitney *U* test while controlling for type I error using the Bonferroni adjustment. Data were represented as boxplots. All statistical analyses were performed using the SPSS version 23 (IBM, Chicago, IL, USA). *p* < 0.05 was considered statistically significant.

## Results

### Study population

In total, 520 IVDs were analyzed from the 104 volunteers. The mean body mass index (BMI) was 23.2 ± 3.2 kg/m^2^ (ranged 16.2–33.2), and there were 196 (38.7%), 140 (26.9%), 95 (18.3%), 66 (12.7%), and 23 (4.4%) IVDs in each of the Pfirrmann classification from grade I to V, respectively (Table 2). The remaining 497 IVDs of Pfirrmann I–IV were quantitatively analyzed.

**Table 2:** Participants’ characteristics

Characteristics	Number of cases (%)
Gender	
Male	47 (45.2)
Female	57 (54.8)
BMI (kg/m^2^)	23.2
Intervertebral disc	520
Lumbar vertebral body	624
Pfirrmann grading	
I	196 (38.7)
II	140 (26.9)
III	95 (18.3)
IV	66 (12.7)
V	23 (4.4)
Age (years)	
20–29	27 (26.0)
30–39	17 (16.3)
40–49	17 (16.3)
50–59	26 (25.0)
60–70	17 (16.3)

**Fig. 2:**
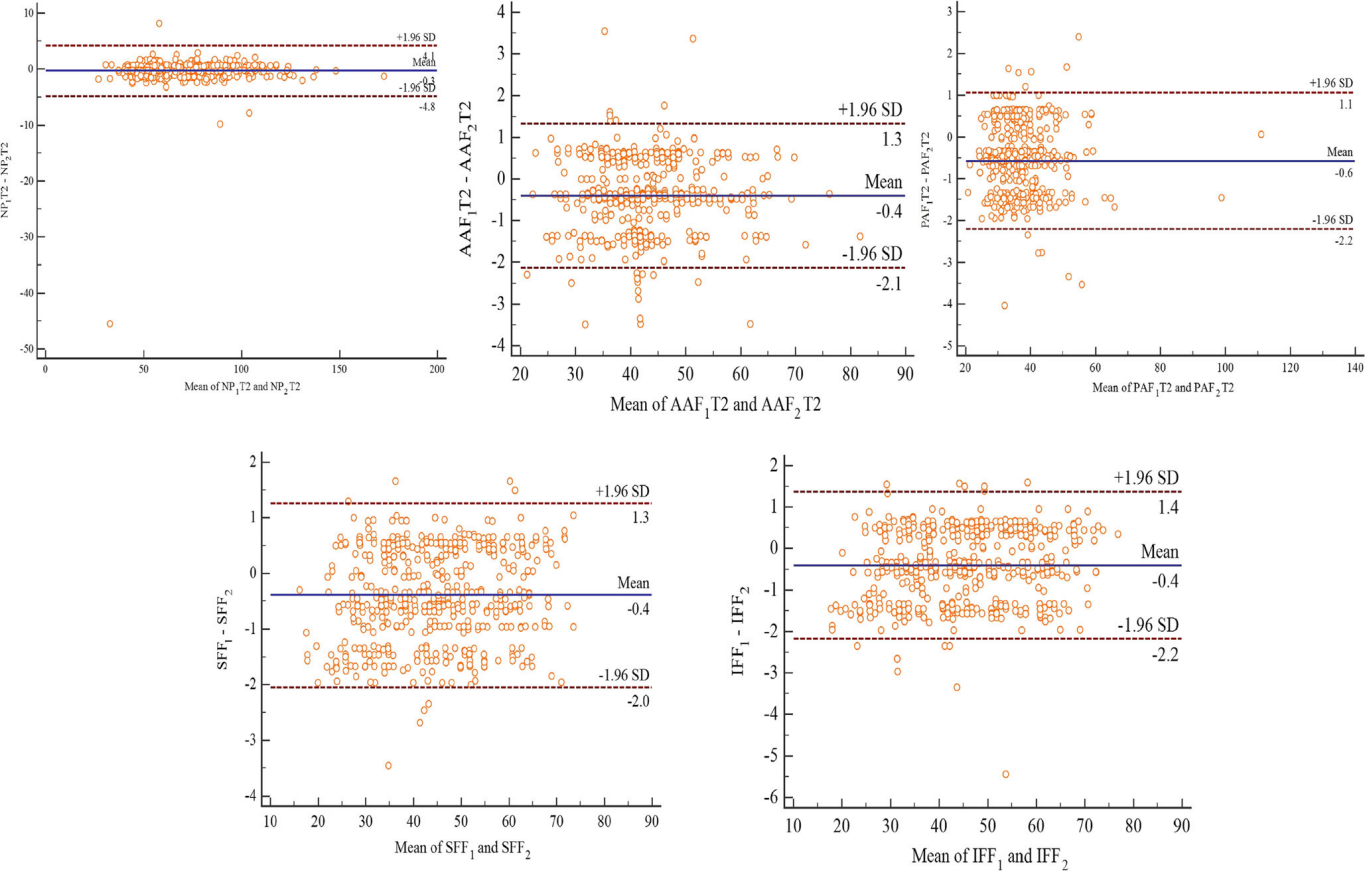
Bland–Altman plots comparing all paired measurements, including NP, AAF, PAF, SFF, and IFF. Bias (solid line) and limits of agreement (dashed line) are shown for each variable. The mean score is plotted on the *x*-axis, while the difference between the two devices is plotted on the *y*-axis (mean difference ± 1.96 SD). SFF, superior vertebral fat fraction; IFF, inferior vertebral fat fraction; NP, nucleus pulposus; AAF, anterior annulus fibrosus; PAF, posterior annulus fibrosus

Pfirrmann classification of *κ* = 0.83 demonstrated an excellent agreement, while Bland–Altman analysis of all paired measurements revealed a mean difference (± SD, 95% limits of agreement) of − 0.3 ms (± 2.3 ms, − 4.8 to 4.1 ms) for NP; − 0.4 ms (± 0.9 ms, − 0.5 to − 0.3 ms) for AAF; − 0.6 ms (± 0.8 ms, − 0.7 to − 0.5 ms) for PAF; − 0.4 ms (± 0.8 ms, − 0.5 to − 0.3 mmHg) for SFF; and − 0.4 ms (± 0.9 ms, − 0.5 to − 0.3 mmHg) for IFF as shown in Fig. 2.

### Correlations of VFF and T2 in AAF, NP, PAF/ Pfirrmann grading in all discs, and lumbar vertebral bodies

**Fig. 3:**
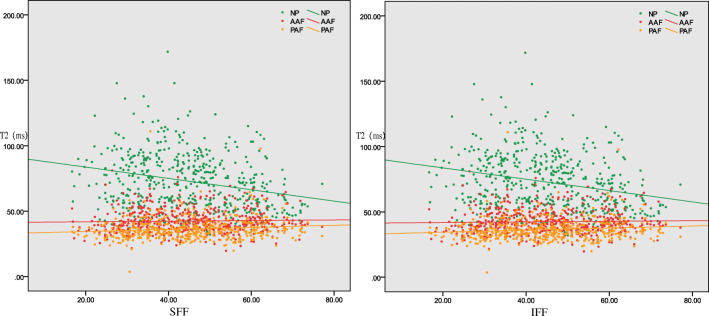
Correlation between all the T2 values of annulus fibrosus and nucleus pulposus and the FF values of adjacent superior and inferior vertebrae together. The T2 values of the NP and AAF and PAF significantly correlated with the FF values of the adjacent superior and inferior vertebral bodies (*p* < 0.001). FF, fat fraction; SFF, superior vertebral fat fraction; IFF, inferior vertebral fat fraction; NP, nucleus pulposus; AAF, anterior annulus fibrosus; PAF, posterior annulus fibrosus

**Fig. 4:**
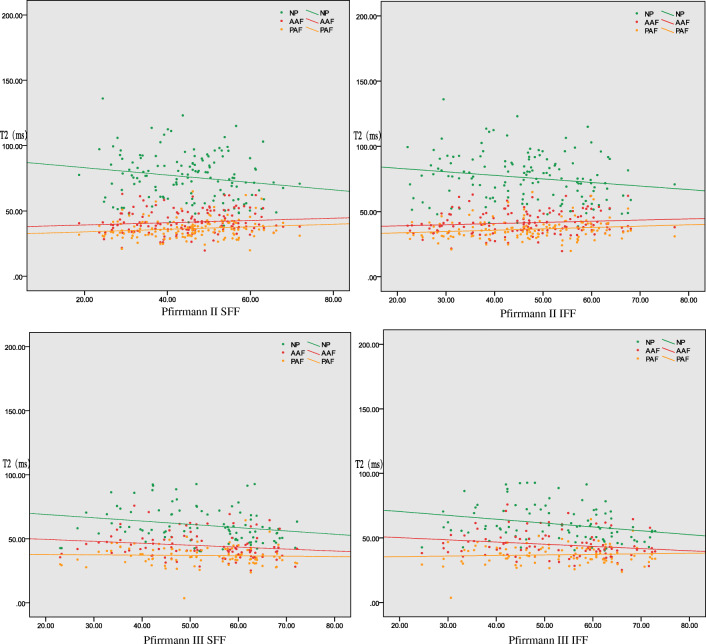
Correlation between the T2 values of annulus fibrosus and nucleus pulposus in different Pfirrmann grades and the FF values of adjacent superior and inferior vertebrae. The T2 values of the NP of grade II and III significantly correlated with the FF values of the adjacent superior and inferior vertebral bodies (*p* < 0.05), while the other T2 values of the NP and annulus fibrosus did not significantly correlate with the FF values of the adjacent upper and lower vertebral bodies (*p* > 0.05). FF, fat fraction; SFF, superior vertebral fat fraction; IFF, inferior vertebral fat fraction; NP, nucleus pulposus; AAF, anterior annulus fibrosus; PAF, posterior annulus fibrosus

**Fig. 5:**
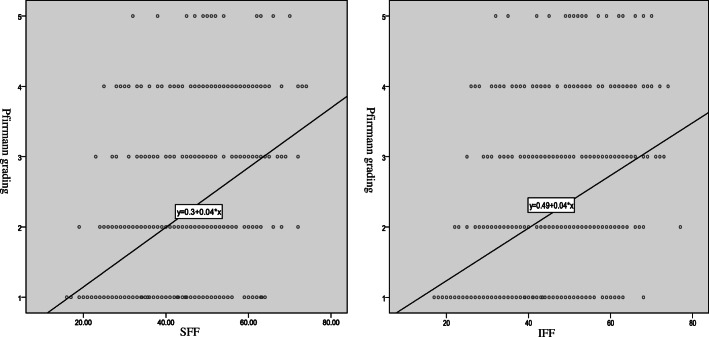
Different Pfirrmann grades of intervertebral discs correlated significantly with VFF values in the superior vertebrae and inferior vertebrae (*p* < 0.01). VFF, vertebral fat fraction; SFF, superior vertebral fat fraction; IFF, inferior vertebral fat fraction

For all 497 discs, the T2 value of the NP/AAF/PAF significantly correlated with not only the SFF but also the IFF (*p* < 0.001, Fig. 3). The NP T2 was negatively correlated with adjacent VFF (*p* < 0.001, Fig. 4). The Pfirrmann classification I–V correlated positively with the adjacent SFF and IFF (*p* < 0.01) (Fig. 5).

### Correlations and differences of VFF and T2 in AAF, NP, PAF/Pfirrmann grading in the four Pfirrmann groups

**Fig. 6:**
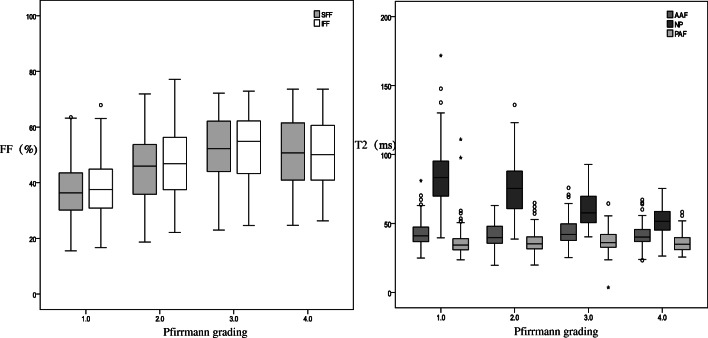
Box chart of different Pfirrmann-graded disc NP and annulus fibrosus T2 values of the upper and lower adjacent vertebral body FF values. NP T2 values are decreasing gradually but FF values are inversely increasing only in Pfirrmann I–III. FF, fat fraction; SFF, superior vertebral fat fraction; IFF, inferior vertebral fat fraction; NP, nucleus pulposus; AAF, anterior annulus fibrosus; PAF, posterior annulus fibrosus

There was no statistical difference between SFF and IFF adjacent to all the IVDs (*p* > 0.05). However, only the T2 values of NP significantly inversely correlated with the adjacent VFF in Pfirrmann classification degrees II–III but not in grades IV–V (Fig. 3). When comparing II–III and IV parameters, significant differences were observed in the NP T2 values and SFF, but none was found in the AAF T2, PAF T2, adjacent IFF, ages, gender, and the BMI parameters (Tables 3 and 4). NP T2 values decreased gradually from Pfirrmann I to IV grades, while FF values increased in Pfirrmann I–III and decreased in Pfirrmann IV (Fig. 6).

**Table 3:** Differences of VFF and T2 in AAF, NP, and PAF in the four Pfirrmann groups

Pfirrmann grade	T2			SFF	IFF
	NP	AAF	PAF		
I	83.9 ± 20.5	42.5 ± 8.7	36.0 ± 9.5	37.1 ± 9.8	38.4 ± 10.3
II	76.2 ± 19.1	41.6 ± 8.8	36.6 ± 7.5	45.0 ± 11.3	46.3 ± 12.0
III	60.6 ± 13.2	44.4 ± 10.1	37.0 ± 7.7	52.1 ± 11.8	52.5 ± 12.0
IV	52.2 ± 10.2	41.1 ± 9.2	36.2 ± 6.7	50.5 ± 12.7	49.9 ± 12.7
*χ*2	161.983	7.048	4.291	111.970	93.933
*p*	0.000	0.070	0.0232	0.000	0.000

*SFF*, superior vertebral fat fraction; *IFF*, inferior vertebral fat fraction; *NP*, nucleus pulposus; *AAF*, anterior annulus fibrosus; *PAF*, posterior annulus fibrosus

**Table 4:** Differences of II–III and IV–V in Mann–Whitney *U* test

II–III vs IV	T2			SFF	IFF	Ages	Gender	BMI
	NP	AAF	PAF					
Z	− 7.264	− 1.044	− 0.565	− 1.496	− 0.585	− 1.714	− 0.065	− 0.962
*p*	0.000	0.297	0.572	0.135	0.559	0.086	0.948	0.336

*SFF*, superior vertebral fat fraction; *IFF*, inferior vertebral fat fraction; *NP*, nucleus pulposus; *AAF*, anterior annulus fibrosus; *PAF*, posterior annulus fibrosus

### Correlations and differences of VFF and T2 in AAF, NP, and PAF/Pfirrmann grading in the five age groups

**Fig. 7:**
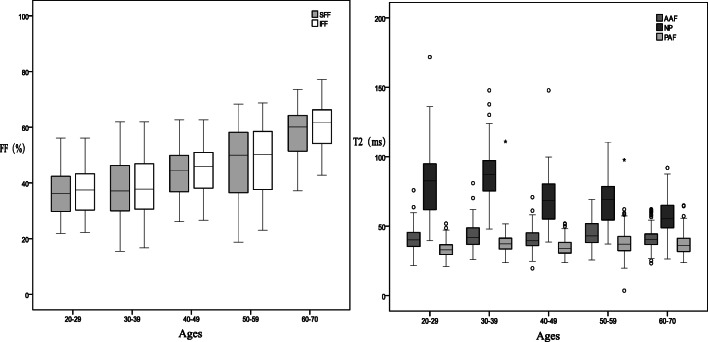
FF values of the vertebral body increased with age, while the T2 value of the NP decreased after age 30. After age 50, FF increases more rapidly than normal. FF, fat fraction; SFF, superior vertebral fat fraction; IFF, inferior vertebral fat fraction; NP, nucleus pulposus; AAF, anterior annulus fibrosus; PAF, posterior annulus fibrosus

Quantitative MRI characteristics were assessed for age groups: 20–29 years, 30–39 years, 40–49 years, 50–59 years, and 60–70 years (Fig. 7). T2 values of NP were weakly correlated with the adjacent VFF values in the age group 40–49 years (*r* = 0.246). The VFF value increased with age while the NP T2 value decreased after the age of 30. After the age of 50, VFF increases more rapidly. The NP T2 value and VFF of adjacent vertebrae bone were significantly different between the age groups 30–39 and 40–49, and the age groups 50–59 and 60–70 (*p* < 0.001) (Table 5).

**Table 5:** Differences of VFF and T2 in AAF, NP, and PAF in the five age groups

Ages	T2			SFF	IFF
	NP	AAF	PAF		
20–29	80.9 ± 22.9	41.0 ± 8.1	33.4 ± 5.1	36.2 ± 7.7	36.9 ± 7.6
30–39	86.6 ± 20.0	43.3 ± 9.6	38.5 ± 10.0	37.8 ± 11.6	38.3 ± 11.8
40–49	68.0 ± 17.8	40.6 ± 8.2	35.1 ± 6.0	44.0 ± 9.2	45.1 ± 9.1
50–59	67.5 ± 17.1	45.0 ± 10.0	38.6 ± 10.2	47.9 ± 12.0	48.6 ± 12.2
60–70	57.1 ± 12.4	42.0 ± 9.2	37.2 ± 7.8	58.6 ± 8.9	60.3 ± 8.4
*χ*2	110.852	14.379	41.747	175.239	186.836
*p*	0.000	0.003	0.000	0.000	0.000

*SFF*, superior vertebral fat fraction; *IFF*, inferior vertebral fat fraction; *NP*, nucleus pulposus; *AAF*, anterior annulus fibrosus; *PAF*, posterior annulus fibrosus

### Correlations and differences of VFF and T2 in AAF, NP, and PAF/Pfirrmann grading in the five lumbar levels

**Fig. 8:**
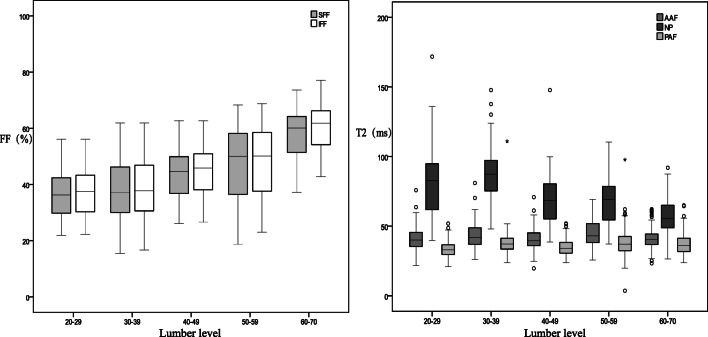
Different lumbar levels of disc nucleus pulposus and annulus fibrosus T2 values of the inferior and superior adjacent vertebral body FF values. The T2 value decreased and the FF value increased from L1 to L5 levels. FF, fat fraction; SFF, superior vertebral fat fraction; IFF, inferior vertebral fat fraction; NP, nucleus pulposus; AAF, anterior annulus fibrosus; PAF, posterior annulus fibrosus

**Fig. 9:**
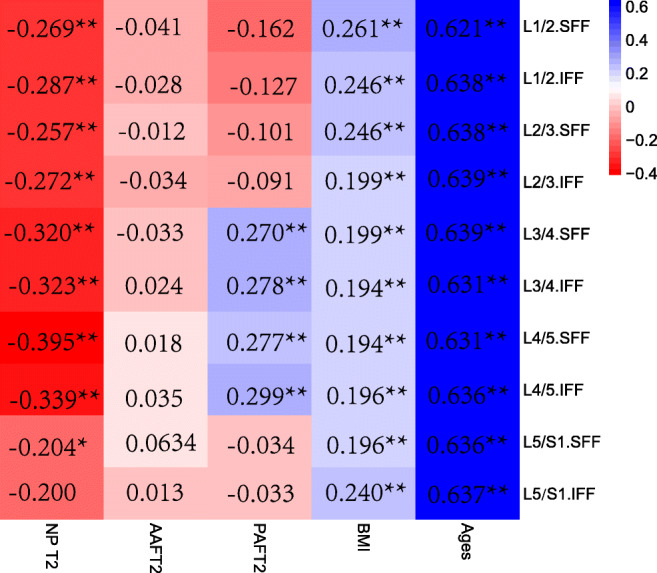
Correlations of FF with NP T2 values, annulus fibrosus T2 values, BMI, and ages in different lumbar levels. *p < 0.05; **p < 0.01. Values marked with an asterisk indicate statistical significance. FF, fat fraction; SFF, superior vertebral fat fraction; IFF, inferior vertebral fat fraction; NP, nucleus pulposus; AAF, anterior annulus fibrosus; PAF, posterior annulus fibrosus

We observed a relationship not only between the NP T2 value and adjacent VFF but also between the Pfirrmann grading and adjacent VFF in L1/2-L5/S1 levels, which was statistically significant except for the L5/S1 NP T2 value and the S1 VFF. However, the AAF and PAF did not significantly correlate to VFF (Figs. 8 and 9). T2 value of NP, AAF, and PAF and its adjacent SFF were significantly difference from those of L1/2 to L5/S1 (Table 6). Among the 104 volunteers, the number of IVDD (Pfirrmann II–V IVD) occurring alone in one segment was 8 and contained the following: L1/2 (*n* = 0), L2/3 (*n* = 0), L4/5 (*n* = 4), L3/4 (*n* = 2), and L5/S1 (*n* = 2).

**Table 6:** Differences of VFF and T2 in AAF, NP, and PAF in the five lumbar levels

	T2			SFF	IFF
	NP	AAF	PAF		
L1/2	72.6 ± 19.3	40.3 ± 8.4	39.0 ± 12.3	40.1 ± 11.3	43.3 ± 12.4
L2/3	76.0 ± 20.4	43.9 ± 9.5	37.5 ± 6.3	43.2 ± 12.5	45.6 ± 12.8
L3/4	76.6 ± 20.4	45.3 ± 9.0	37.0 ± 6.5	45.6 ± 12.7	45.4 ± 12.7
L4/5	72.3 ± 21.5	44.2 ± 8.8	35.4 ± 6.1	44.8 ± 12.9	45.4 ± 12.9
L5/S1	67.6 ± 20.6	38.4 ± 8.2	33.0 ± 7.2	46.2 ± 12.9	44.6 ± 13.1
*χ*2	12.184	45.154	36.957	13.601	1.900
*p*	0.016	0.000	0.000	0.006	0.754

*SFF*, superior vertebral fat fraction; *IFF*, inferior vertebral fat fraction; *NP*, nucleus pulposus; *AAF*, anterior annulus fibrosus; *PAF*, posterior annulus fibrosus

## Discussion

We investigated whether IVDD is associated with adjacent vertebrae BMF using advanced quantitative MRI techniques. We found clear correlation between the T2 of NP/AAF/PAF and adjacent VFF when we put all the intervertebral discs and vertebral bodies together. Furthermore, according to the Pfirrmann classification standard, we proposed grade I as representing a normal disc, grade II and III as representing the early stages of disc degeneration, and grade IV as representing the advanced stages of disc degeneration. The NP T2 values of early degenerated IVD (grade II–III) are greater than those advanced (grade IV). Our study revealed that only the NP T2 value of early degenerative disc correlated mildly with the adjacent vertebrae BMF content and differed significantly between the early and the advanced degenerative groups. These results showed that it is the early stages of the IVDD and the BMF, and the IVDD can influence each other at some level.

According to the moderate correlation between Pfirrmann grading and VFF, the significance of VFF in both correlation analyses with the NP T2 value and discrimination between the advanced degenerated groups was minimal in our study and may limit the clinical relevance. The small sample size of Pfirrmann IV (12.7%) with severe IVDD might be a possible explanation. Since IVDD and BMF may be affected by age as well as BMI, the presence of elderly or overweight individuals may have influenced the results. In Pfirrmann V, we were unable to determine whether there was a correlation or not due to the measurement error associated with collapsed disc height.

A moderate to high inverse correlation has been observed between the BMF (mean and SD) and T1ρ/T2 (mean and SD) in the adjacent disc of most lumbar levels by Vangeneugden et al. [27]. Similarly, they also have reported a significant correlation between BMF SD and adjacent disc Pfirrmann classification, with a correlation coefficient of 0.53 [27]. We reconfirmed that the severity of disc degeneration increased with the increase of fat in adjacent vertebral bodies, and this relationship is more pronounced in L4/5 lumbar levels. The number of IVDD occurring alone in one segment was highest in the L4/5, suggesting that L4/5 IVD may be the most vulnerable segment. Fullerenol nanoparticles, as free radical scavengers, may prevent vertebral fatty marrow deposition and inflammatory responses during disc degeneration [28]. Disc height is associated with increased vertebral fracture risk in postmenopausal women [13]. The BMF can, therefore, directly or indirectly change the disc’s microstructure and its biochemical composition. This study confirmed the role of vertebral BMF in lumbar disc degeneration and provides further evidence in the currently limited field.

MRI quantitative and semi-quantitative studies have been conducted for patients with various ages with a primary focus on the variation of IVDD [29] or BMF [30], modic changes [31], and T2 and FF parameters studied separately with increasing age. To our knowledge, most studies have not reported the correlation of IVD T2 value and the Pfirrmann grading parameters with BMF in different ages. In age groups 40–49, NP T2 value was found to be weakly inversely correlated with adjacent BMF, while other age groups appeared to demonstrate no statistically significant correlation. This indicates that the most rapidly changing phase (the disc degeneration and fat conversion speed) is the same as the relationship between Pfirrmann II–III.

Our study demonstrated significant associations between the biochemical degeneration of IVD and adjacent vertebrae BMF using advanced MRI quantitative techniques such as IDEAL-IQ and T2 mapping, as well as MRI biomarkers sensitive and objective to collagen integrity and water content of the disc [32]. We found some compositional changes in the disc and bone marrow, which occurred earlier than the visually observable morphologic changes with conventional MRI sequences. Consequently, quantitative measurement of vertebral BMF content has potential predictive value in early disc degeneration.

Our study had several limitations. First, our study is a cross-sectional cohort study, and a longitudinal study needs to be carried out to obtain further evidence. Second, our results may be limited since it was only based on the relationship between T2 mapping and BMF, and perfusion factors associated with cellular and microvascular density were not evaluated. However, Karampinos et al. confirmed that there is significant correlation between reduced vertebral bone marrow perfusion indices and osteoporosis using dynamic contrast-enhanced MRI [33]. Additionally, transversal T2 mapping scanning with a 3-mm layer thickness may have various partial effects on the adjacent end plates due to disc space narrowing. Thus, adding a sagittal plane view in future studies may provide more information.

In conclusion, our results demonstrate that there is a close association between BMF and IVDD, and that a reciprocity relationship may exist between the vertebrae BMF and the disc nutrition supply. Novel quantitative measurement of BMF deposition and vertebral disc water content have potential predictive value and provide important supplements to the qualitative traditional classification strategies for the early stages of IVDD. However, the suggested causality needs to be further investigated and confirmed in larger longitudinal studies.

## Abbreviations

QMRI: Quantitative magnetic resonance imaging
IDEAL-IQ: Iterative decomposition of water and fat with echo asymmetry and least squares estimation
BMF: Bone marrow fat
IVD: Intervertebral disc
IVDD: Intervertebral disc degeneration
FF: Fat fraction
VFF: Vertebral fat fraction (bone marrow fat fraction of the vertebra)
SFF: Superior vertebral fat fraction
IFF: Inferior vertebral fat fraction
NP: Nucleus pulposus
AAF: Anterior annulus fibrosus
PAF: Posterior annulus fibrosus
